# Sexual Orientation and Functional Pain in U.S. Young Adults: The Mediating Role of Childhood Abuse

**DOI:** 10.1371/journal.pone.0054702

**Published:** 2013-01-23

**Authors:** Andrea L. Roberts, Margaret Rosario, Heather L. Corliss, David Wypij, Jenifer R. Lightdale, S. Bryn Austin

**Affiliations:** 1 Department of Society, Human Development, and Health, Harvard School of Public Health, Boston, Massachusetts, United States of America; 2 Department of Psychology, City University of New York, The City College and Graduate Center, New York, New York, United States of America; 3 Division of Adolescent and Young Adult Medicine, Boston Children’s Hospital, Boston, Massachusetts, United States of America; 4 Department of Pediatrics, Harvard Medical School, Boston, Massachusetts, United States of America; 5 Channing Division of Network Medicine, Brigham and Women’s Hospital, Harvard Medical School, Boston, Massachusetts, United States of America; 6 Department of Biostatistics, Harvard School of Public Health, Boston, Massachusetts, United States of America; 7 Department of Cardiology, Boston Children’s Hospital, Massachusetts, United States of America; 8 Division of Gastroenterology and Nutrition, Boston Children’s Hospital, Boston, Massachusetts, United States of America; Catholic University of Sacred Heart of Rome, Italy

## Abstract

**Objective:**

Pain without known pathology, termed “functional pain,” causes much school absenteeism, medication usage, and medical visits. Yet which adolescents are at risk is not well understood. Functional pain has been linked to childhood abuse, and sexual orientation minority youth (gay, lesbian, bisexual, “mostly heterosexual,” and heterosexual with same-sex sexual contact) are more likely to be victims of childhood abuse than heterosexuals, thus may be at greater risk of functional pain.

**Methods:**

We examined sexual orientation differences in past-year prevalence of functional headache, pelvic, and abdominal pain and multiple sites of pain in 9,864 young adults (mean age = 23 years) from a large U.S. cohort. We examined whether childhood abuse accounted for possible increased risk of functional pain in sexual minority youth.

**Results:**

Sexual minority youth, except for gays and lesbians, were at higher risk of functional pelvic and abdominal pain and multiple sites of pain than heterosexuals. Gay and lesbian youth had elevated prevalence only of abdominal pain. Childhood abuse accounted for 14% to 33% of increased experience of multiple sites of pain in minority youth.

**Conclusions:**

Youth who identify as “mostly heterosexual” or bisexual or who identify as heterosexual and have had same-sex partners comprised 18% of our sample. Clinicians should be aware that patients with these orientations are at elevated risk of functional pain and may be in need of treatment for sequelae of childhood abuse. Conventional categorization of sexual orientation as heterosexual or homosexual may fail to distinguish a large number of youth who do not wholly identify with either group and may be at elevated risk of health problems.

## Introduction

Pain without known pathology is termed “functional pain” and can include recurrent abdominal pain, headache, backache, pelvic and musculoskeletal pain. Functional pain causes much impairment, disability, and medical care usage. In children, functional pain is associated with school absenteeism [1], medication usage, and medical visits [2]. Patients with multiple medically unexplained pains and symptoms incur more than twice the medical expenses of patients without such symptoms. Extrapolated to the whole U.S., these elevated expenses amount to $256 billion excess medical costs annually attributable to functional pain and symptoms [3]. Thus, identifying groups at increased risk for functional pain and gaining better understanding about possible causes of functional pain in those groups is of great public health interest.

In this paper we examine whether sexual minority young adults report more functional pain, including headache, abdominal pain, and pelvic pain, than heterosexuals in a large U.S. cohort, the Growing Up Today Study (GUTS). Dysregulation of the hypothalamic-pituitary-adrenal system and related inflammatory processes resulting from abuse or violence victimization may predispose individuals to experience functional pain [4]. Sexual orientation minorities are exposed to more childhood abuse [5], bullying, harassment [6], and crime victimization [7] than heterosexuals, for multiple reasons: intolerance toward gender nonconforming behavior, which is more prevalent among sexual orientation minorities [8], [9], anti-gay violence following revealing one’s identity as gay, lesbian, or bisexual, and minorities’ increased participation in risk-taking behaviors resulting from internalized stigmatization of their sexual orientation [10]. Although exposure to violence is a major risk factor for functional pain and is more prevalent in sexual minorities, it is largely unknown whether sexual orientation disparities exist in prevalence of functional pain.

In the present paper, we examine functional pain in the head, abdomen and pelvis by sexual orientation. As prior research indicates that experience of more types of functional pain and other functional symptoms is associated with greater and more persistent impairment, we also examine prevalence of multiple sites of functional pain [11]. We examine potential differences in pain across a broad spectrum of sexual orientation, including youth who identify as “mostly heterosexual” and youth who identify as heterosexual and have had same-sex sexual partners. Although “mostly heterosexuals” comprise the largest group of sexual minority youth, their health risks are not well understood [9], [12], [13]. Additionally, prior research has found that heterosexuals with any lifetime same-sex sexual partners report higher exposure to childhood abuse compared to heterosexuals with no lifetime same-sex sexual partners, thus, may also be at higher risk of experiencing functional pain [5], [14]. We further examine whether disparities in childhood abuse partly account for possible sexual-orientation disparities in pain. Finally, we conduct analyses comparing siblings discordant on sexual orientation to control for family-level factors, measured or unmeasured, that affect risk of pain.

## Methods

### Sample

We use data from the Growing Up Today Study, a U.S. longitudinal cohort of 16,882 children of women participating in the Nurses’ Health Study II, established in 1996 and followed up annually or biennially [15]. This paper reports data primarily from the 2007 wave, when respondents were 19 to 27 years old (mean age 22.7 years), which assessed experience of pain at three sites, sexual orientation, and childhood maltreatment (n = 9864). The Institutional Review Board at Brigham and Women’s Hospital approved this study.

### Measures

#### Pain

We queried experience of pain at three sites: head, abdomen, and pelvis. Presence of recurrent headaches was assessed with three questions. Respondents were first asked “How often do you have headaches?” with response options: never, 1–2 times per year, 3–6 times, 7–11 times, 12–24 times, or more than 24 times per year. Respondents were then asked if when they had a headache they had difficulty doing normal activities (bed rest necessary) or if pain prevented them from doing routine activities. Respondents were considered to have impairing headaches if they experienced them 7–11 times per year or more and had difficulty with routine activities. Respondents were further asked “Do you only have headaches after drinking alcohol?” Respondents who answered “yes” to this question were considered not to have functional headaches. Because there is not a clear precedent in the literature for a frequency requirement for impairing headaches, we conducted sensitivity analyses with frequency dichotomized at 3–6 times per year or more versus fewer than 3 times per year and at 12–24 times per year or more versus fewer than 12 times per year.

We defined functional abdominal and pelvic pain as pain not associated with medically diagnosed conditions (i.e., Crohn’s disease). To distinguish abdominal pain from pelvic pain the questionnaire included a figure of a human torso with the area around the navel shaded to indicate pain locations considered abdominal or “belly” and the area around the pelvis and genitals shaded to indicate pain locations considered pelvic. Based on preferred terminology determined from a pilot study, the pelvic area was labeled “the pelvis” for females and “the groin” for males.

Functional abdominal pain was assessed with a question about the frequency of pain in the past year, with response options: never, 1–2 times per year, 3–11 times per year, monthly but not weekly, weekly but not daily, or daily. Respondents were then asked when they had abdominal pain, how difficult it made going to school or work, from 0: no difficulty to 6: extreme difficulty. Respondents were also asked how difficult the pain made it for them to participate in social or recreational activities with the same response scale, adapted from the Interference subscale of the Multidimensional Pain Inventory [16]. In alignment with Rome III criteria for functional dyspepsia or abdominal migraine [17], functional abdominal pain was defined as present if the respondent experienced pain monthly or more often or scored as 3 or higher on their difficulty in going to work or school or engaging in social or recreational activities. Functional pelvic pain was assessed with similar questions regarding pain in the genitals/pelvis or genitals/groin.

When considering pain experienced in either the abdomen or pelvis, respondents were asked to exclude pain from menstrual cramps, surgery, pregnancy, childbirth, sports-related injury, food poisoning, and stomach flu. Respondents were asked whether they had received a health care provider’s diagnosis for their pain, and if so, what it was. Respondents who had received a non-functional diagnosis such as esophageal tear or testicular torsion were considered not to have functional pain in the relevant area.

As experience of multiple functional pains or symptoms have been associated with more impairment and increased health care usage, we examined experience of pain in multiple sites [3]. Experience of pain in multiple sites was coded present if the respondent experienced pain in two or three sites and absent if the respondent experienced no pain or pain in one site; 930 respondents (8.7%) were missing pain information.

*Sexual orientation* was assessed with two questions. The first asked whether during their lifetime respondents had had sexual contact with males, females, both, or no sexual contact [18]. The second question asked “Which of the following best describes your feelings? (1) completely heterosexual (attracted to persons of the opposite sex), (2) mostly heterosexual, (3) bisexual (equally attracted to men and women), (4) mostly homosexual, (5) completely homosexual (gay/lesbian, attracted to persons of the same sex), or (6) unsure.” [19] People who reported “completely heterosexual” feelings and same-sex sexual contact were categorized as “heterosexual with same-sex contact.” “Mostly homosexual” and “completely homosexual” responses were collapsed to form a “lesbian/gay” category because individual category sizes were small; people “unsure” of their feelings were excluded from analyses (n = 3, 0.03%).

#### Childhood abuse

Respondents were asked about childhood abuse during two time periods: before age 11 years and ages 11–17 years. Physical and emotional abuse in each time period was measured with the Physical/Emotional Abuse Subscale of the Childhood Trauma Questionnaire [20]. Four questions were asked about frequency of adults in the family: yelling and screaming, saying hurtful or insulting things, punishing in a way that seemed cruel, and hitting hard enough to leave bruises or marks. Each item was coded from 0 (never) to 4 (very often), and a score was formed from the sum following coding recommendations [20].

Experiences of sexual abuse occurring when the responded was a child (before age 11 years) and a teen (ages 11 to 17 years) were measured separately with two questions: one that asked the respondent about being touched by or forced to touch an adult or older child in a sexual way when s/he did not want to, and a second question that asked about an adult or older child forcing or attempting to force sexual activity by threatening, holding down, or hurting the respondent [21]. Sexual abuse was coded separately as present or absent for the child and teen years. Among respondents providing sexual orientation and pain information, 5 were missing one or more abuse responses.

#### Covariates

Age at time of questionnaire return was continuous; race/ethnicity was coded as White or not White.

### Analyses

To establish whether functional headache, abdominal and pelvic pain as well as the prevalence of pain in two or more sites differed by sexual orientation, we examined the prevalence of experience of these pains by sexual orientation, separately for males and females. To ascertain whether sexual orientation was associated with presence of functional pain adjusted for covariates, we modeled each type of pain and pain in two or more sites as the dependent variable with sexual orientation as the independent variable. We tested a sex-by-sexual-orientation interaction term in these models to examine whether these relationships varied by sex.

In a prior publication, we documented higher exposure to childhood abuse among sexual orientation minorities in this cohort [14]. To investigate whether this higher exposure to childhood abuse partly accounted for a possible relationship between sexual orientation and experience of pain in multiple sites, we created an additional model with abuse added to the model. We calculated the mediation proportion for this model using the publicly available Mediate macro [22]. The mediation proportion based on our analyses represents the proportion of excess pain experienced by sexual orientation minorities relative to heterosexuals statistically accounted for by exposure to childhood abuse.

Because our cohort includes multiple families with more than one sibling enrolled, we were also able to examine pain differences within families and between siblings discordant for sexual orientation. These additional analyses account for factors shared by siblings, both measured and unmeasured, that may increase risk for pain, including family dysfunction, modeling of pain by parents, and genetic susceptibility [23]. We conducted conditional logistic regression models conditioned on family, with multiple sites of pain as the dependent variable and sexual orientation as the independent variable, using SAS PROC PHREG [24]. For these models we coded sexual orientation dichotomously as heterosexual with no same-sex contact versus all others, to limit comparisons to heterosexual siblings versus sexual-minority siblings rather than examining comparisons among sexual-minority siblings. In these models, only families in which two siblings were discordant for experience of multiple sites of pain can contribute to effect estimates.

For all other models, we used generalized estimating equations to account for possible clustering of data in families, using SAS 9.2 [24]. To test for differences in prevalence of outcomes by sexual orientation, we used a binomial distribution with a log link. To estimate risk ratios (RR) with our dichotomous dependent variables, we specified a Poisson distribution with a log link [25]. With the exception of tests for significance of prevalence differences, models were adjusted for race, age at questionnaire completion, and sex.

Respondents with sexual orientation data and any measures of pain or childhood abuse were included in reports of prevalence (n = 9784, 6143 women, 3641 men). Eighty respondents missing sexual orientation data were excluded (0.8%). These respondents were more likely to be male (66.3% versus 37.2%, p<0.001) and were older than included respondents (mean = 23.6 versus 23.2 years, p<0.05), but did not differ on race. Respondents with complete data on pain and childhood abuse were included in models (n = 8884, 5672 women, 3212 men). Respondents excluded from models (n = 980, 9.9%) were more likely to be male (49.2% versus 36.2%, p<0.001) and were older (mean = 23.8 versus 23.1 years, p<0.001) than included respondents, but did not differ on race.

## Results

Each type of functional pain and experience of multiple sites of pain were more prevalent in “mostly heterosexual” youth compared with heterosexuals with no same-sex sexual contact (the reference group) (Table 1). Abdominal and pelvic functional pain and multiple sites of pain were more prevalent in bisexual females and heterosexual females with same-sex sexual contact. Females had approximately 2 to 3 times the prevalence of headache, functional abdominal and pelvic pain, and multiple sites of pain than males.

**Table 1:** Functional pain by sexual orientation, Growing Up Today Study (n = 9,784).†

	Heterosexual (n = 7828)	Heterosexual, same-sex sexual contact (n = 171)	Mostly heterosexual (n = 1417)	Bisexual (n = 172)	Gay/lesbian (n = 196)
	%				
**Functional pain**					
**Headache**					
Females	25.8	32.2	31.0***	30.2	26.8
Males	10.7	12.5	15.8**	16.0	14.3
**Abdominal**					
Females	17.2	25.0*	25.0***	29.5***	24.1
Males	6.5	12.5	13.5***	12.0	9.5
**Genital/pelvic/groin**					
Females	6.4	17.2***	10.4***	18.6***	8.4
Males	2.5	7.5	5.5**	8.0	1.9
**Two or more sites of pain**					
Females	8.5	21.6***	15.4***	20.9***	9.6
Males	2.3	5.0	6.6***	8.0	3.8

† N is smaller for some rows due to missing values.

* Statistically significant Wald χ^2^,

* = p<.05,

** = p<.01,

*** = p<.001 compared with heterosexuals with no lifetime same-sex sexual contact.

In models of each type of pain and multiple functional pains, heterosexuals with same-sex contact, mostly heterosexuals, and bisexuals were at higher risk of abdominal, pelvic, and multiple pains (RR range: 1.5 to 3.0) (Table 2). Sexual orientation disparities in risk of headache were smaller, with only mostly heterosexuals at statistically significantly increased risk. Results were nearly identical in sensitivity analyses with functional headache dichotomized at 3–6 headaches per year or at 12–24 headaches per year. Interestingly, lesbian and gay youth were not at increased risk of headache, pelvic pain, or multiple pains, though they had elevated risk of abdominal pain. The sex-by-sexual-orientation interaction term was not statistically significant in these models.

**Table 2:** Risk ratios of past-year experience of functional pain by sexual orientation using generalized estimating equations, Growing Up Today Study (n = 9,784).†

	Headache	Abdominal pain	Pelvic pain	Multiple sites of functional pain
	Risk ratio [95% confidence interval]			
**Sexual orientation**				
Heterosexual (n = 7828)	1.0 [Reference]			
Heterosexual, same-sex sexual contact (n = 171)	1.2 [1.0, 1.6]	1.5 [1.1, 2.1]**	2.7 [1.8, 4.0]***	2.5 [1.8, 3.5]***
Mostly heterosexual (n = 1417)	1.2 [1.1, 1.4]***	1.5 [1.4, 1.7]***	1.7 [1.4, 2.0]***	1.9 [1.6, 2.2]***
Bisexual (n = 172)	1.2 [0.9, 1.6]	1.7 [1.3, 2.3]***	3.0 [2.1, 4.3]***	2.6 [1.8, 3.6]***
Gay/lesbian (n = 196)	1.1 [0.9, 1.5]	1.4 [1.0, 2.0]*	1.1 [0.6, 2.2]	1.2 [0.7, 2.2]

† Adjusted for sex, age at questionnaire return, and race/ethnicity.

Wald χ^2^ test significant at:

* = p<0.05;

** = p<0.01;

*** = p<0.001.

Sexual and physical/emotional abuse experienced as a teenager were significant predictors of experiencing multiple sites of functional pain; exposure to sexual and physical/emotional abuse as a child (through age 11 years) was not significantly associated with multiple sites of pain after adjusting for abuse in the teen years. Higher prevalence of exposure to abuse partially mediated elevated prevalence of multiple sites of pain in sexual orientation minorities (mediation proportion range, 14 to 33%) (Table 3, Model 2). Mediation was highest in bisexuals (33%) and lowest in heterosexuals with same-sex sexual contact (14%).

**Table 3:** Risk ratios of past-year experience of functional pain in two or more sites by sexual orientation, with mediation by childhood abuse, using generalized estimating equations, Growing Up Today Study (n = 8,907).†

	Model 1: Sexual orientation (Base model)	Model 2: Sexual orientationand childhood abuse	Mediation proportion, abuse (Model 2)
	Risk ratio [95% CI]		%
Sexual orientation			
Heterosexual (n = 7426)	1.0 [Reference]		
Heterosexual, same-sex sexual contact (n = 162)	2.5 [1.8, 3.5]***	2.2 [1.6, 3.1]***	14**
Mostly heterosexual (n = 1357)	1.9 [1.6, 2.2]***	1.6 [1.3, 1.9]***	28***
Bisexual (n = 163)	2.5 [1.8, 3.6]***	1.9 [1.3, 2.7]*	33***
Gay/lesbian (n = 191)	1.2 [0.7, 2.1]	1.0 [0.6, 1.8]	N/A
Childhood abuse			
Child sexual abuse		1.0 [0.9, 1.3]	
Teen sexual abuse		1.7 [1.4, 2.1] ***	
Child physical/emotional abuse		1.0 [0.6, 1.7]	
Teen physical/emotional abuse		3.2 [2.0, 5.2]***	

† Adjusted for sex, age at questionnaire return, and race/ethnicity.

Wald χ^2^ test significant at:

* = p<0.05;

** = p<0.01;

*** = p<0.001.

CI: Confidence interval. N/A: Not applicable: gay men and lesbians were not at significantly higher risk of multiple sites of pain, therefore there is no elevated risk to explain in mediation analyses.

Because child abuse was strongly associated with functional pain, we conducted additional analyses to estimate prevalence of experiences of childhood abuse in persons reporting multiple sites of pain. Almost half of respondents with multiple sites of pain (47%) reported sexual abuse or the highest quartile of physical/emotional abuse in either childhood or adolescence, compared to 24% of respondents who reported no functional pain (not in table).

In analyses examining between-sibling differences in risk of experiencing multiple sites of functional pain (n = 351 individuals), the estimated effect of sexual orientation minority status was attenuated 30%, from RR = 2.0 (95% CI = 1.7, 2.3; p<.001) to RR = 1.7 (95% CI = 1.0, 2.9; p<.05), suggesting that family clustering of factors that influence experience of pain may partly confound the estimated effects of sexual orientation on pain risk in the whole sample.

## Discussion

Our principal finding is that sexual-minority youth are at higher risk of functional headache, pelvic, and abdominal pain as well as experience of multiple sites of pain and that these pain disparities are partially explained by higher exposure to childhood abuse, particularly during adolescence, compared with heterosexuals. Youth who identified as “mostly heterosexual” comprised 14% of our sample (1 in 7 participants) and had approximately twice the prevalence of multiple sites of pain than heterosexuals. Clinicians, including neurologists, gastroenterologists, gynecologists and urologists, should be aware that youths with this orientation are at risk for functional pain and may be in need of treatment for sequelae of childhood abuse. Conventional categorization of sexual orientation as heterosexual or homosexual may fail to distinguish a large number of youth who do not fully identify with either group and who may be at elevated risk of health problems.

Unexpectedly, youth who identified as mostly or completely homosexual were an exception to the pattern of elevated risk of pain in sexual minorities. Gay and lesbian youth did not have statistically significantly elevated prevalence of two of the three functional pains we examined or of multiple sites of pain, despite having greater exposure to sexual abuse in childhood compared with the reference group, as previously reported [14].

We are aware of only one report that has examined possible sexual orientation differences in experience of pain. A study of California residents ages 18 to 72 years asked separately about experience of headaches or migraines, back problems, and any chronic pain. In this study an increased prevalence of headaches or migraines was identified among gay men and heterosexual men with male sexual partners, and higher prevalence of back problems was found among bisexual women and heterosexual women and men with same-sex sexual partners compared with heterosexuals without same-sex partners. When examining chronic pain, however, the study did not find significantly higher prevalence among sexual-orientation minorities [26]. Unlike the California study, we did not find statistically significantly elevated prevalence of headache among gay men or heterosexual men with same-sex sexual partners. Our results may have differed from the results in this study due to different specification of head pain (functional headaches in our study versus any headache or migraine in the California study) or due to differences in the characteristics of the sample, for example, our sample had a mean age of 22 years, while only 22% of the California sample was younger than age 29 years.

Prior studies suggest that differences in social support and positive orientation-group identity among gay and lesbian youth may be an explanation for the differences in experience of pain we found among sexual minority groups. For persons with same-sex attraction or history of same-sex sexual contact, identification as gay or lesbian versus bisexual, mostly heterosexual, or heterosexual may provide a positive group identity and increased social support that is protective of experience of functional pain. In one study of older gay, lesbian, and bisexual persons, participants were more satisfied with social support received from those who knew their sexual orientation than from those who did not know their orientation and that satisfaction with social support was associated with reduced loneliness [27]. Studies have also indicated that identifying as bisexual may be particularly challenging in terms of providing connection to the sexual-minority community, certainty about sexual orientation identity, and orientation disclosure [28]. Lack of social support for bisexual identity has also been associated with internalized negativity towards a bisexual identity [29].

Persons identified as “mostly heterosexual” represented the largest group of sexual minorities in our sample. The few studies that have examined this orientation group suggest that they may experience substantial health disparities compared to heterosexuals [30]. To our knowledge only one study has examined social support in mostly heterosexuals. This study of urban female youth found “mostly heterosexuals” reported receiving significantly lower social support from both family and friends than did heterosexuals [31]. Because issues related to sexual orientation identity have not been examined in this group, the extent to which orientation identity poses challenges is unknown. Additionally, it may be that identifying as gay or lesbian indicates an acceptance of minority orientation that is protective against orientation-related stressors and therefore reduces experience of functional pain. Further investigation to clarify these relationships is warranted.

The extent to which childhood abuse statistically accounted for increased pain varied considerably among sexual minority groups. These factors accounted for 28% and 33% of the elevated pain in mostly heterosexuals and bisexuals respectively, but only 14% of elevated pain in persons identified as completely heterosexual but with a history of same-sex sexual contact; this finding suggests important heterogeneity of factors contributing to the elevated pain experienced by subgroups of sexual minorities.

Our finding of elevated pain risk in sexual minorities persisted in analyses comparing sexual minorities with their heterosexual siblings, but pain differences were attenuated. Heterosexual siblings of sexual minorities in our sample experienced some, but not all, of the elevated risk for functional pain that minorities experienced. This finding provides evidence that some of the elevated risk for functional pain experienced by sexual minorities is at the family level. One possibility is that heterosexual siblings witnessed or heard about abuse or harassment of their sexual-minority sibling and these stressful experiences increased their own risk of multiple pains. Because some of the risk for functional pain was at the family level, studies seeking to identify causal risk factors for functional pain should consider family factors as well as assessing possible confounding by family-level factors in estimating effects of individual-level risk factors.

Our findings should be considered in light of four limitations. First, childhood abuse was assessed retrospectively; therefore recall error could bias estimates. Second, sexual-orientation minorities may be more willing to report stigmatizing information, such as abuse victimization histories, than heterosexuals, which would inflate estimates of the association between orientation and abuse. Third, we did not conduct physical exams; therefore some people included as having functional pain may have had medically explained (non-functional) conditions. Fourth, questions regarding same-sex sexual experiences did not specify consensual sexual experiences. As sex-abuse perpetrators are overwhelmingly male [32], some men reporting heterosexual orientation and same-sex sexual experiences may not have had any consensual same-sex experiences.

In this large sample of U.S. young adults, report of multiple functional pains was strongly associated with sexual minority group status, with the exception of being gay or lesbian. Reporting multiple pains was also strongly associated with reports of childhood abuse. Of respondents with two or three sites of pain, almost half reported exposure to at least one type of childhood abuse (47%).

Patients with child abuse history rarely discuss this history with their clinician, thus, clinicians must assume the responsibility for broaching this critical topic [33]. Identification of pain as functional is crucial for successful treatment because a variety of treatments, including cognitive behavioral therapy, psychotherapy, and pharmaceuticals targeting the central nervous system have been found to be effective for functional disorders [34].
